# The Treatment of Some Common War Neuroses

**Published:** 1918-03

**Authors:** 


***The Treatment of Some Common War Neuroses.*** By E. D. ADRIAN and L. R. YELLAND. Abs. by J. M. WOLFSOHN from *The Lancet*, June 1917.

In their communication the authors have presented a most valuable method of procedure in the treatment of hysterical conditions. They point out that the stigma attached to the word " hysteria " is not fair to the patient, because hysteria is usually held to mean that there is nothing wrong and that no treatment is required, but, as Yelland says, " how absurd is this attitude in the case of a man who has been stone deaf for months — his disability is real enough to him and he is in urgent need of treatment ". The term functional, rather than hysterical, is objected to on the ground that the former term covers too wide a field and would include such disorders as neurasthenia and epilepsy; so for the present hysteria is the best term to use.

The paper gives the result of treatment of 250 cases of common types of hysteria, including 88 cases of mutism, 34 of deafness, 18 of aphonia, 37 monoplegias, 46 paraplegias, 16 hemiplegias, 18 disordered gaits, not associated with organic change. In 95 o/o of the cases the treatment instituted has been immediately *successful.*

Because of its present intense interest the discussion and procedure used is given in rather full detail. In pre-war days, one had the idea that when the diagnosis of hysteria was established, the task of the physician was at an end; now, not only diagnosis, but adequate treatment is essential, in order to make the difference between a useless burden to the state and a useful citizen, and even an adequate soldier.

The authors insist that a correct or rather a confident diagnosis is the first step and the most important part of the treatment. They say: " When properly carried out it is extremely rapid and almost invariably successful, even in cases which have been treated for months and years without result ".

They do not use this treatment in the exhausted neurasthenic type of patient who complains of headache, insomnia, etc.; but only in the type of neurosis where the disability is some hysteric symptom, e. g., paralysis, loss of speech, etc. The chief phenomena underlying the hysterical type of mind are weakness of the will and intellect (as shown by the Binet Simon mentality test, and either personal or family instability); negativism which may be due either to a severe inertia, or to an active but not necessarily conscious resistance to the idea of recovery; and the contraction of the antagonistic groups of muscles on attempted movement, and hypersuggestibility, as shown by production of suggestive symptoms, for example, anesthesia, contraction of the visual fields, etc. The unconscious resistance must be broken down before any results can be obtained.

In considering treatment and progress one must take into account:

(1) The fixed idea which gives rise to the functional symptom, e. g., loss of voice, etc.

(2) The state of mind which allowed the fixed idea to develop.

The former can be treated by suggestive methods, but the latter will not vanish and the patient will always be liable to develop hysterical troubles in moments of emotional stress and exhaustion. As Pitres said, " En réalité, on naît hystérique, on ne le devient pas ".

Psycho-analysis and its methods are taken up, but dismissed quickly, because it is felt that suggestion plays a large part in the cure when the treatment is successful and that the psycho-analysis, when seriously applied, requires too long a time to be of practical use, in order to purge the mind of all the accumulated filth of a lifetime. The treatment used is aimed only at relieving the functional symptoms.

Three principles are employed (1) suggestion, (2) re-education, (3) discipline. In other words, suggestion makes the patient believe he will be cured and from this springs the belief that he is cured. Re-education brings the disordered functions back to normal and the bad habit is lost. Discipline breaks down the unconscious resistance of the patient to the idea of recovery. Some adopt the watchful waiting attitude, which, in the opinion of the authors, is not justified, as the environment of hospital life is distinctly enervating.

Hypnotism in the hands of the authors has led them to believe it to be too slow and uncertain in comparison with vigorous suggestive treatment and re-education. It acts better with subjective troubles, such as insomnia, dreams, etc., which are scarcely hysterical. Strict isolation on the other hand is an important adjunct to their treatment as it makes the patient's illness an unprofitable, dreary business; especially is this true in functional tremors and pseudo-chorea, as the patient becomes quiet when deprived of his audience.

Under re-education, simple persuasion or the process by which the patient is convinced by logical argument that he is not so ill as he supposes, one must be on one's guard not to let the patient be under the impression that he is suspected of malingering. The treatment which has given the best results in the authors' hands is very brief suggestion, followed by rapid re-education, which is continued without pause until the function is entirely regained.

Where discipline is needed isolation is not such a desirable form of treatment, as it takes too much time and is only as effective as a little plain speaking accompanied by a strong faradic current. 8 hours' rest out of 24 is enough in these cases, as an extension of that time for one who has no sign of physical illness, is liable to do more harm than good.

The disciplinary element is necessary in some cases, as it is the only certain means of breaking down the patient's resistance to the idea of recovery.

The following general considerations hold good in every case :

(1) It is essential that the patient be convinced that immediate recovery will be produced. This conviction can be strengthened by using a form of treatment which is capable by itself of evoking some part of the function which is in abeyance, e. g., tickling the back of the throat in a case of mutism, in order to produce reflex phonation. If there be too much resistance on the part of the patient, a faradic current can be made strong enough to break down the unconscious barriers to sensation in the most profound fonctional anesthesias. Electricity unaccompanied by necessary suggestion is usually innocuous, cases treated by this means alone usually foster in the patient the idea that his case is hopeless. The sole value of the electric treatment lies in the suggestive effect it can be made to produce.

The chief element in the cure must be that the physician understands the patient's case and is able to cure him; which idea should be fostered from the moment the patient enters the ward. The case is investigated as briefly as possible, each symptom is accepted as normal under the circumstances, and not interesting or obscure. If the case is to be demonstrated, it is shown as a perfect example of a case which is cured in five minutes by appropriate treatment. After examination the patient is left with assurance that the physician will cure him as soon as he finds time to do so. It is always better to ascertain what previous treatment the patient has had. Do not discuss the case or the methods to be used before the patient, nor allow the patient to air his views on the subject.

The treatment should be carried out in a special room where nothing will distract the patient's attention. Reeducation is commenced with the appearance of the first sign of recovery before the patient can collect his thoughts; command and persuasion combined until the disordered function has completely recovered. *Rapidity* and authoritative manner are the chief factors in the re-education and before the patient is allowed to go back to the wards complete recovery should be aimed at. If treatment has to be discontinued, he is told that he will be quite well in 24 hours and re-education should be continued as soon as possible.

The authors now take up the common hysterical symptoms, namely, disorders of speaking and hearing as the most frequent. By this method 33 out of 34 cases of hysterical deafness were cured after a few minutes treatment of each. *Mutism* associated with deafness, should be treated after the deafness has been cured. Yelland uses stimulation with a pharyngeal electrode for this purpose and with the first signs of voluntary phonation the patient should be given no respite until he can speak perfectly. *Aphonia* is usually the effect of laryngitis, though it may be a stage in the recovery of mutism and is treated by a tickling of the palate, or gargling.

*Stammering* is harder to treat and requires intensive re-education. *Hysterical blindness* in military cases is rare, but monocular or defective vision with no objective signs are more often malingering. Successful treatment by strong faradism in the region of the supra-orbital nerves, together with a little plain speaking, is very efficacious.

*The paralysis of the upper limbs.* — Hysterical brachial monoplegia is very common after a slight wound or bruise necessitating a splint. Those cases which show an anesthesia are easy to treat because after the cure of the anaesthesia, it is simple to cure the paralysis; rapid re-education is then employed until the normal function is restored. Monoplegia of the lower limb is treated in the same way as the above mentioned. Recent hysterical paraplegias, due to shell shock, are flaccid with normal reflexes; if recovery takes place slowly it merges into the spastic type, with pseudo-clonus, exaggerated reflexes and rigidity, with strong contracture of the antagonistic group of muscles, which is entirely of mental origin.

The authors mention some minor points which deserve emphasis.

(1) Slippers without heels should not be used during re-education, as they encourage a shuffling gait.

(2) No sticks should be used which might perpetuate a limp.

(3) The use of a large room, or corridor, is necessary for re-educational treatment, as it gives a certain privacy. As little assistance as possible is given during re-educational treatment. The patient should walk arm in arm with the physician, then facing him, holding both his hands, then one hand only, and finally with no support whatever. A fall should never be taken as an excuse for resting.

The same form of treatment should be used in all functional gaits, functional sciaticas, rheumatism, and paraplegias. The more serious the disability, the easier the cure.

*Tremors, fits, etc.*

Tremors are harder to cure than the above mentioned disabilities. In these cases suggestive measures, in the inducing and curing of fits, are usually not permanent but serve more largely as an aid to diagnosis than to treatment.

Involuntary movements and hysterical chorea yield to isolation treatment better than suggestion. Truly hysterical contractures yield at once to suggestive faradism, they point out that one must be careful before treatment to ascertain that these are not the reflex disorders described by Babinski and Froment, which are as yet generally intractable.

General anesthesia is not much used by these authors, because the method demands an anesthetist, who can produce a long excitement stage, and the results to some extent are a matter of luck, and failure by this means makes other methods useless.

The cause of the production of the neurosis determines the disposition in most of the cases. Most of them are recommended for home duty. If, after severe strain and exhaustion, a neurosis appears, a short rest is perhaps all that is necessary. Relapses may occur in these cases but subsequent cures are more easily accomplished.

The authors conclude by making a plea for a thorough examination of the past history, and of the mental condition, before deciding what form of service will be most advantageous to the patient and to the nation. In the more intractable conditions it is advantageous to return the patient to civilian life[1].

1. Since reading this article, the reviewer has seen one of the authors treat several cases by the above named means, with startling results.

*Le Gérant* : O. Porée. Paris — Imp. Lahure, 9, rue de Fleurus.

